# Modeling of partial dome collapse of La Soufrière of Guadeloupe volcano: implications for hazard assessment and monitoring

**DOI:** 10.1038/s41598-019-49507-0

**Published:** 2019-09-11

**Authors:** Marc Peruzzetto, Jean-Christophe Komorowski, Anne Le Friant, Marina Rosas-Carbajal, Anne Mangeney, Yoann Legendre

**Affiliations:** 1 Université de Paris, Institut de physique du globe de Paris, CNRS, F-75005 Paris, France; 2 BRGM, Orléans, France; 3 BRGM, Guadeloupe, France

**Keywords:** Natural hazards, Solid Earth sciences, Mathematics and computing

## Abstract

Over the past 9,150 years, at least 9 flank collapses have been identified in the history of La Soufrière of Guadeloupe volcano. On account of the volcano’s current unrest, the possibility of such a flank collapse should not be dismissed in assessing hazards for future eruptive magmatic as well as non-magmatic scenarios. We combine morphological and geophysical data to identify seven unstable structures (volumes ranging from 1 × 10^6^ m^3^ to 100 × 10^6^ m^3^), including one that has a volume compatible with the last recorded flank collapse in 1530 CE. We model their dynamics and emplacement with the SHALTOP numerical model and a simple Coulomb friction law. The best-fit friction coefficient to reproduce the 1530 CE event is tan(7°) = 0.13, suggesting the transformation of the debris avalanche into a debris flow, which is confirmed by the texture of mapped deposits. Various friction angles are tested to investigate less water-rich and less mobile avalanches. The most densely populated areas of Saint-Claude and Basse-Terre, and an area of Gourbeyre south of the Palmiste ridge, are primarily exposed in the case of the more voluminous and mobile flank collapse scenarios considered. However, topography has a prominent role in controlling flow dynamics, with barrier effects and multiple channels. Classical mobility indicators, such as the Heim’s ratio, are thus not adequate for a comprehensive hazard analysis.

## La Soufrière of Guadeloupe volcano

The Guadeloupe archipelago is located in the northern part of the Lesser Antilles arc that resulted from subduction of the North and South American plates under the Caribbean plate. This process initiated volcanism about 40 Ma ago^1^. Activity of the inner arc in the last 3 Ma built seven volcanic complexes on the island of Basse-Terre (Guadeloupe), progressing from north to south^2–5^.

**Figure 1:**
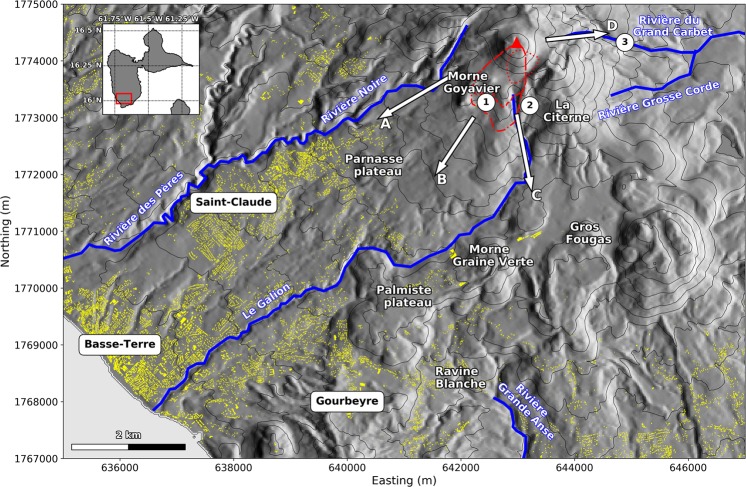
Rivers and main topographic features around La Soufrière of Guadeloupe volcano (red triangle), along with the three main cities (Saint-Claude, Basse-Terre and Gourbeyre). The upper left insert features the Guadeloupe island, with the red rectangle matching the extent of the map. ① Ravine des Bains Jaunes, ② Ravine de la Citerne, ③ Second Carbet waterfall. Arrows A, B, C and D identify the main flow pathways discussed in the main body of the text. Yellow patches are buildings (data from IGN BDAlti). The lateral extent of some initial unstable volumes is displayed with the red lines. Dashed-dotted line: *topA2* and *midA2* scenarii; dashed line: *topA1_inf* and *topA1_sup* scenarii; dotted line: *dolomieu* scenario. The DEM is from IGN BDTopo, coordinates: WGS84, UTM20N. The contour interval is 100 m.

La Soufrière of Guadeloupe is an andesitic active volcano. It belongs to the 0.445 Ma old Grande Découverte-La Soufrière volcanic complex^2,3^ and is located about 2 km north of the town of Saint-Claude where about 10 000 people live (Fig. 1). Successive eruptions and erosion phases built a complex and steep landscape (see Fig. 1 for the following geographic names). To the south and south-west, old massive lava flows (Parnasse Plateau, Palmiste plateau) and eruptive centers (Morne Goyavier, La Citerne, Morne Graine Verte, Gros Fougas) partially protect inhabited areas. They are cut by numerous ravines, such as the Ravine de la Citerne and the Ravine Blanche. Three main rivers have their source in the vicinity of La Soufrière volcano: Le Galion and Rivière Noire flow south-west twoards the cities of Saint-Claude and Basse-terre, and the Rivière du Grand Carbet heads east.

At least 15 Holocene magmatic eruptions (9 lava dome eruptions and 6 explosive plinian to sub-plinian eruptions) have been identified. Phreatic and hydrothermal activity is also recurrent, along with partial edifice collapses. Over the last 9, 150 years, at least 9 debris-avalanches occurred, mainly to the south-west, and reached a distance of 9–15 km from the dome^3,6–8^. The last magmatic eruption, in 1530 CE, started with a partial flank-collapse of 80 ± 40 × 10^6^ m^3^. It then produced sub-plinian tephra fallout, a lava dome, and pyroclastic density currents from column and dome collapse^7–10^. In addition, recent studies have shown that a small magmatic eruption occured in 1657 ± 20 years Cal. CE^8^. Since 1635 CE, 6 phreatic explosions have been witnessed^2,3,11,12^. The most recent and violent one took place in 1976–1977 and led to the evacuation of more than 70 000 people^2,3,11,13^. It may have been triggered by a small intrusion of magma that did not reach the surface^11,14–16^.

La Soufrière is monitored by the Guadeloupe Volcanological and Seismological Observatory (OVSG-IPGP), and has shown over the last two decades an increasing unrest^3,16–18^. Shallow seismicity has been progressively increasing, as has the temperature of some acid-sulfate thermal springs^14,16–18^. Fumarolic activity has also strengthened, leading to a partial restriction of access to the dome in 1999^3^. In February and April 2018, three seismic swarms mainly composed of hybrid volcano-tectonic earthquakes released a total seismic energy of about 90 GJ^17–20^. Such an energy release had not been measured for 40 years. Furthermore, near-field deformations, including inflation (3–7 mm/year) and flank basal spreading (7–10 mm/year), are recorded^17–20^.

The past history of La Soufrière volcano of Guadeloupe, its structure, its deformation, its well-developed hydrothermal system, and the current activity constitute factors that favor a future instability, as observed on many other volcanoes^21^. Thus, we are concerned with the consequences of slope failure involving the current lava dome. Instability could be significantly enhanced as a result of magma or hydrothermal pressurisation^22^, intense volcanic seismicity, a strong local-to-regional earthquake (e.g. 21 st November 2004 Mw = 6,3 earthquake^23^), or extreme rainfall (Casita-style collapse^24^). Such failure could trigger rock avalanches or debris avalanches depending on material water content^25^. Mixing of hydrothermal fluids^3,12^ with the rock avalanches could promote their transition into mobile debris flows if they become saturated and are channelised in ravines. This would significantly enhance their mobility and would engender major risks to population, infrastructure and network, depending on the volume of collapsed material. To address this problem, we investigated different scenarios based on the current geological and geophysical knowledge of La Soufrière of Guadeloupe volcano.

## Dome Structure and Fluid Circulation

**Figure 2:**
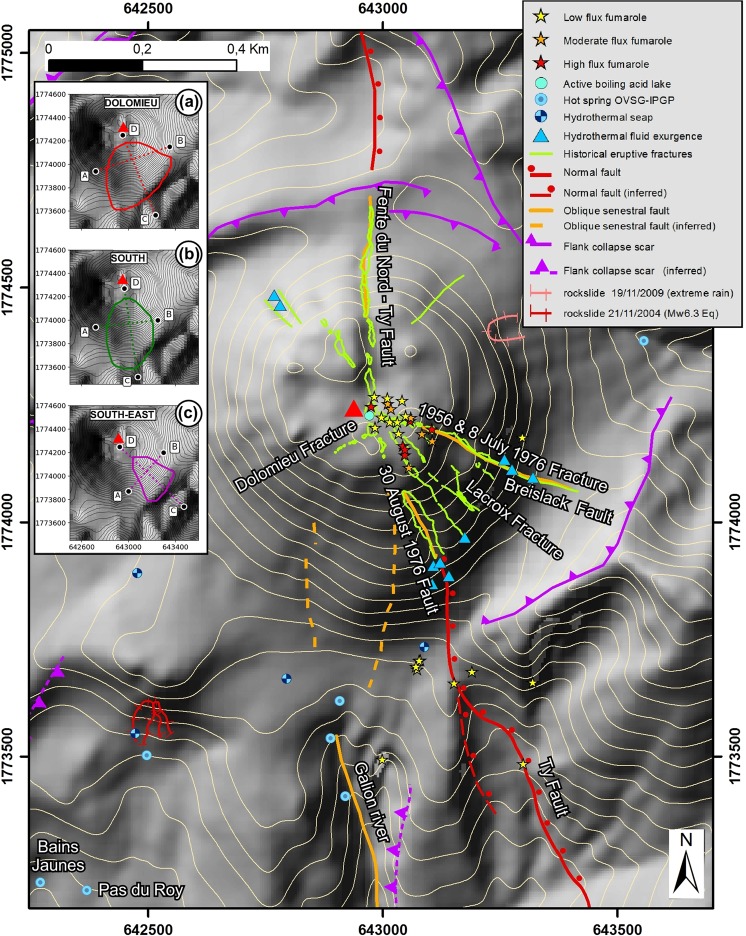
La Soufrière of Guadeloupe lava dome. Main structures of the lava dome and surface manifestations of the active hydrothermal system are displayed. Inserts: (**a**–**c**) are the collapse structure outline for the *dolomieu*, *south*, *south-east* scenarios respectively (Fig. 4). The red triangle is a reference for the center of the dome (Tarissan crater). The DEM is from GeoEye Ikonos 2005-11-25 acquired image processed by Latitude Geosystems, the map was created with the Arcgis software, coordinates: WGS84, UTM20N. The contour interval is 25 m.

Thorough geological surveys have investigated the eruptive history of La Soufrière volcano^3,7–9^. The current dome of La Soufrière (Fig. 2) volcano is composed both of andesite lava and pyroclastic deposits^9,10^. These have been altered over centuries by fluid circulation^26^, and have an average bulk density of 1800 kg/m^3^ ^27^.

Using self-potential measurements, resistivity tomography and density muon radiography, several studies over the last decades have shown the extensive structural and textural heterogeneity of the dome^12,27–33^. Since the last magmatic event, successive phreatic eruptions have led to the creation of numerous fractures (Fig. 2) that constitute major structural discontinuities favouring the circulation of meteoric and acid hydrothermal fluids^16^. The dome is thus divided between dense and relatively unaltered areas, and more fragile parts with active fumaroles and hydrothermal fluid circulation, especially in the south-east of the dome^28,32^.

**Figure 3:**
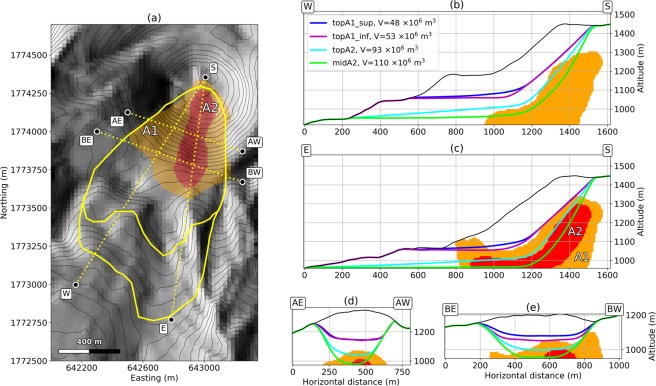
Collapse geometries of deep rooted scenarios. The inner yellow contour in (**a**) is the extent of *topA1_sup* and *topA1_inf* scenarios, the outer yellow contour is the extent of *topA2* and *midA2* scenarii. In (**b**–**e**), longitudinal and transverse cross-sections of the initial (black line) and post-collapse (colored lines) topographies are given. The A1^12^ conducting body (orange area) and A2^12^ conducting body (red area) are seen from above in (**a**), and within cross-sections in (**b**–**e**). Cross-sections extents and directions are given by the corresponding letters S, W, E, AE, AW, BE, BW in (**a**). The DEM is from IGN BDTopo, coordinates: WGS84, UTM20N. The contour interval is 20 m.

Perched reservoirs have been identified^12,32^, including one just beneath the fractures opened during the 1976–1977 eruption^11^. This reservoir is the source of fumaroles located along these fractures, and of two acid ponds. It may also be involved in the massive water resurgence that occurred in 1976–1977 and in previous phreatic and hydrothermal historical eruptions^3,12^. The presence of a basal hydrothermalised layer has long been inferred^28,29^. The self-potential positive anomaly in the south-west basal part of the dome identified in previous studies^27^ can be interpreted as structural evidence of the hydrothermal activity linked to the basal layer. Recent 3D electrical tomography^12^ confirms the presence of highly conductive regions inside the dome linked to fluid reservoirs and to the circulation of hot, acidic fluids. The most prominent feature is a massive, listric, conductive body beneath the south-west part of the dome, sloping to the south, with inferred conductivity values higher than 0.1 S.m^−1^ (A1, orange area in Fig. 3). It contains a well defined sub-region with conductivity higher than 1 S.m^−1^ (A2, red area in Fig. 3) starting under the lava dome summit and Tarissan pit, descending south and ending horizontally at the base of the dome where several thermal springs are active in the upper Galion River (Fig. 2). This fluid-saturated and mechanically weakened area can be related to the trend of the SW flank of the dome, that has been shown, by continuous monitoring, to be moving horizontally to the south above the conductive bodies at about 7–10 mm/year^17–20^. We interpret it as basal flank spreading over a decollement surface^12,17–20^. We are concerned that this basal spreading could trigger shallow or deep-seated landslides^34^. The geometry and intrinsic mechanical weakness of these fluid-saturated areas suggest they might be relevant candidates for unstable regions in case of massive partial dome collapse. Indeed, the presence of such a low strength layer at the base of the dome likely contributes to the inherent instability of the edifice^7,12,30,35^. This hypothesis may be supported by the history of Holocene edifice collapse and systematic emplacement of debris avalanches to the south and south- west^3,6–8,36^, that is, in the same direction as the listric, highly conductive bodies A1 and A2.

Along with this active hydrothermal system, the dome is affected by several tectonic active faults (Fig. 2): in particular the Ty fault runs through the dome from the south-east to the north^37,38^. The repeated measurement of the width of the 30^*th*^ August 1976 fracture and of the Fente du Nord (northern section of the Ty fault), using caliper measurements on a 3D metal rod fissurometer, has demonstrated a subsidence and sinistral movement of a few millimetres over the last 35 years on either side of the Ty fault^12,19^. Although the amplitude remains moderate, on the order of 3–10 mm/year^17–19^, these data confirm the potential structural instability of the dome.

## Collapse Scenarios

The stability of volcanic edifices has been thoroughly studied since the dramatic Mount St Helens flank collapse in 1980, but is often hard to assess correctly due to the lack of geotechnical data^22^. Stability is classically studied with Limit Equilibrium Methods, with Finite Element^39^ or Finite Difference^40,41^ numerical schemes. In our case, however, such an analysis is complex due to the lack of data. We therefore define the collapse scars with geometric, geological and geophysical constraints only.

A review of the phenomena associated with 3 and perhaps 4 of the historical non-magmatic hydrothermal eruptions indicates that small collapses within hydrothermally active areas of the dome were associated with small laterally-directed explosions and with rock avalanche flows, with a runout of 1–2 km^3,12,13^. Given the current instability conditions of the dome as well as the current unrest conditions, this scenario of a relatively small destabilization is the most critical and urgent scenario to investigate and to model. However, a more catastrophic destabilization, involving the basal hydrothermal layer, should not be excluded, as it is consistent with past and more voluminous events associated with magmatic eruptions of the last 10 000 years at La Soufrière^3,7,8^.

We thus consider 7 scenarios and summarize their characteristics in Table 1. We first constrain 4 deep-rooted collapse geometries with the main conductive bodies A1 and A2^12^ (Fig. 3). Their lateral extent matches the extent of the A1 body. Their longitudinal profiles feature different shapes: *topA1_sup* (48 × 10^6^ m^3^) follows the top of the A1 conducting body, *topA1_inf* (53 × 10^6^ m^3^) is similar but displays a flatter profile, *topA2* (93 × 10^6^ m^3^) is constrained by the top of the A2 body, and *midA2* (110 × 10^6^ m^3^) cuts through A2.

**Table 1:** Main characteristics and results of the different simulated scenarios.

Scenario name	*south-east*	*south*	*dolomieu*	*topA1_sup*	*topA1_inf*	*topA2*	*midA2*
**Initial Conditions Characterization**							
Scar geometry	superficial			deep-seated			
Most likely forcing	Intense rainfall, earthquake, phreatic eruption			magmatic eruption			
Relative probability of occurrence	high			low			
Volume (×10^6^ m^3^)	1.1	7.1	9.7	48	53	93	110
Proportion in A1	0%			1%	1.5%	8%	11%
Proportion in A2				0%	0%	0.5%	2.5%
Empirical *μ*_*eff*_	0.34	0.29	0.29	0.25	0.25	0.24	0.24
*δ*_*eff*_	18.6°	16.4°	16.0°	14.3°	14.2°	13.6°	13.4°
**Simulation Results**							
Best-fit for the 1530 CE deposits	—			—		*δ* = 7°	—
Material trapped in the scarp	no			yes			
Large lateral spreading of the flow	no	if *δ* ≤ 10°		if *δ* ≤ 10°			
Flow reaches Basse-Terre	no			if *δ* = 7°			
Flow reaches coast	no			no		if *δ* = 7°	
Flow reaches Saint-Claude	no	if *δ* ≤ 10°		if *δ* ≤ 10°			

The proportion of the volume contained in A1 without A2, and in A2, is given in percentage. *μ*_*eff*_ is computed following the Lucas law^52^ *μ*_*eff*_ = *V*^−0.0774^ where *V* is the volume of the unstable material and *μ*_*eff*_ = *tan*(*δ*_*eff*_). In the part of the table “simulation results”, *δ* is the friction angle needed in the simulations to have the result described in the first column.

**Figure 4:**
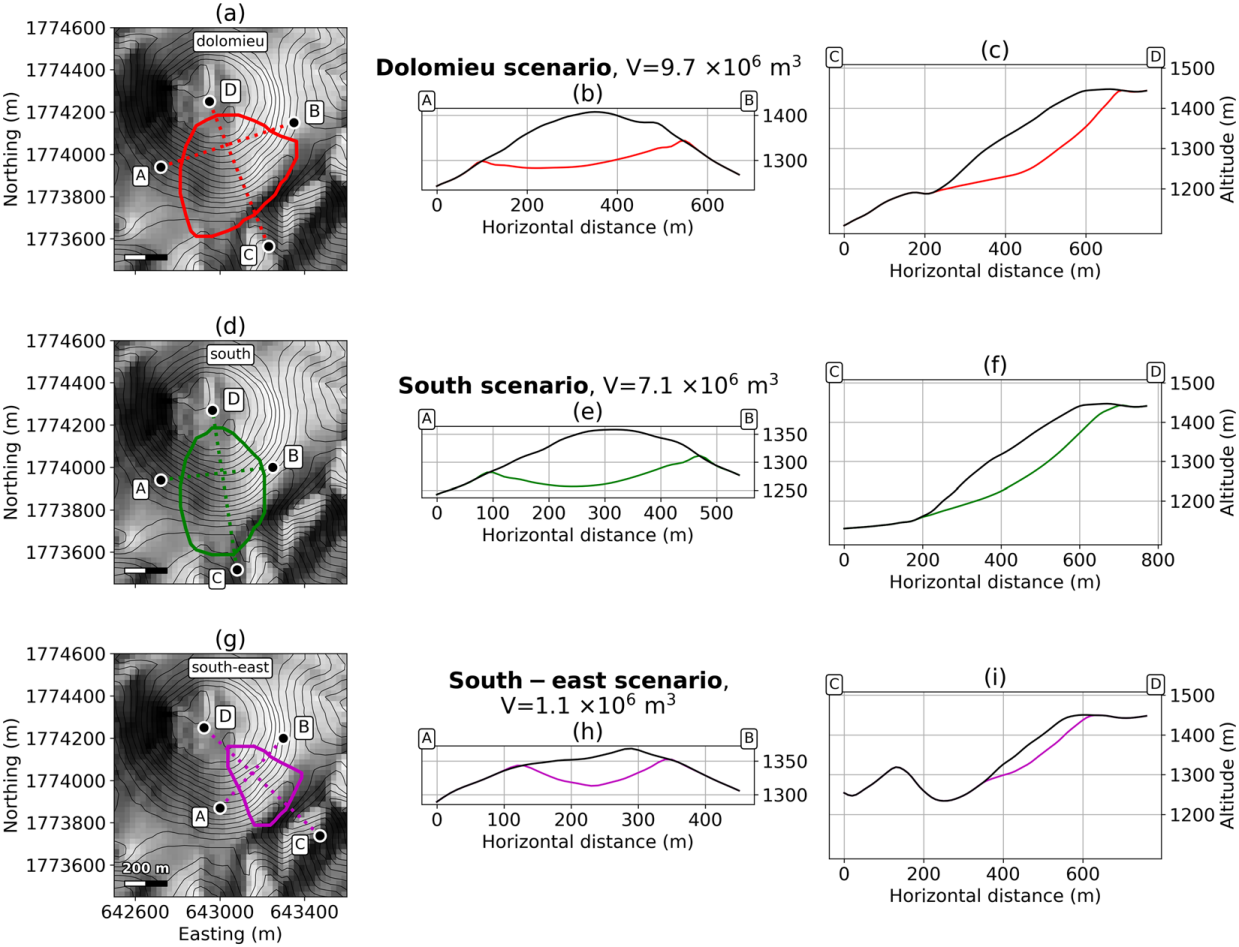
Collapse geometries of superficial scenarios. (**a**,**d** and **g**) respectively display the extent of *dolomieu*, *south* and *south-east* scars. (**b**,**c**,**e**,**f**,**h**,**i**) are crossections of the initial (black line) and post-collapse (colored line) topographies, respectively for *dolomieu*, *south* and *south-east* scenarios. Cross-sections extents are indicated by the letters A, B, C, D. The DEM is from IGN BDTopo, coordinates: WGS84, UTM20N. The contour interval is 20 m.

We then consider 3 superficial geometries for unstable regions with volumes ranging from 1 × 10^6^ m^3^ to 10 × 10^6^ m^3^ (Fig. 4). The *south-east* scenario (1.1 × 10^6^ m^3^) is one of the most plausible scenario as it lies entirely within the area showing the current fumarolic unrest, between the 30^*th*^ August 1976 fault (southern limit), and the 1956–8^*th*^ July 1976 eruptive fractures (northern limit). They are controlled by oblique sinistral fault motion on the Ty and Breislack faults respectively (Fig. 2). The *south* scenario (7.1 × 10^6^ m^3^) extends from the Lacroix fracture to a hypothetical structure linking the Dolomieu fracture to the positive self-potential anomaly identified in previous studies^27^ and discussed previously. The *dolomieu* scenario (9.7 × 10^6^ m^3^) shares this western limit, but goes further to the east to the 1956–8th July 1976 eruptive fractures.

## SHALTOP Numerical Model

In order to simulate the emplacement of the resulting debris avalanche we solve the shallow-water equations, with the main assumptions being that the avalanche is homogeneous and that its thickness is much smaller than its characteristic length. Several numerical models exist to solve these equations, such as Volcflow^42^ and DAN3D^43^, which have been both used to model volcanic flank collapse^42,44^. RAMMS^45,46^ and r.avaflow^47^ numerical models are also commonly used to model debris avalanches and debris flows. In our study we used the SHALTOP numerical model^48–50^ that has already been tested on several natural cases^51–53^ and experiments^50^. It describes a continuous and homogeneous granular flow over a 3D topography. The equations are depth-averaged and numerically solved by taking into account the spatial and temporal variations of the flow thickness and mean velocity, as well as topography curvature. The model calculates the flow thickness in the direction normal to the topography as well as the two-dimensional depth-averaged flow velocity. Processes that would lead to density variations, such as expansion, contraction or incorporation of air and water, are dismissed. Bed erosion is also neglected. Finally, we use a frictional rheology to model the interaction between the flow and the topography, as it has been proven to reproduce the main features of natural landslides^52,54,55^. Furthermore, a previous benchmark of rheological laws^44^ concluded that the frictional rheology yields better results than Bingham or Voellmy rheologies for modeling large volcanic landslides. In depth-averaged models with friction rheologies, the empirical friction coefficient *μ*_*S*_ = *tan*(*δ*), with *δ* the friction angle, can be seen as a phenomenological representation of the dissipation during the flow^50^. It can be constant or depend on the flow thickness and velocity, as for instance in the Pouliquen law^56^. We choose to a use constant friction as it has proved to produce conclusive results^44^, and limits the number of unknown parameters.

For each scenario, simulations are run with various friction angles. We first use a value of 7° that best reproduces the deposits of the 1530 CE event (see following section), and is consistent with previous simulations of dome collapse of La Soufrière of Guadeloupe^36^. This friction angle is typical of debris-flow modeling^55^. It thus corresponds to a highly mobile and mechanically weak material, as characterized for instance the historical non-magmatic volcanic debris avalanches that occurred at Ontake in 1984^57,58^ and Bandai San in 1888^59,60^. In order to investigate drier and less mobile debris avalanches, we also use an empirical relation^52^ relating the friction angle to the volume involved. For our scenarios, the resulting friction angle ranges from 13° to 20°. Finally, we have also considered intermediate values of 10° and 12° to investigate the sensitivity of the simulated deposit to the friction angle.

## Results

### 1530 CE collapse equivalent (*topA2* scenario)

**Figure 5:**
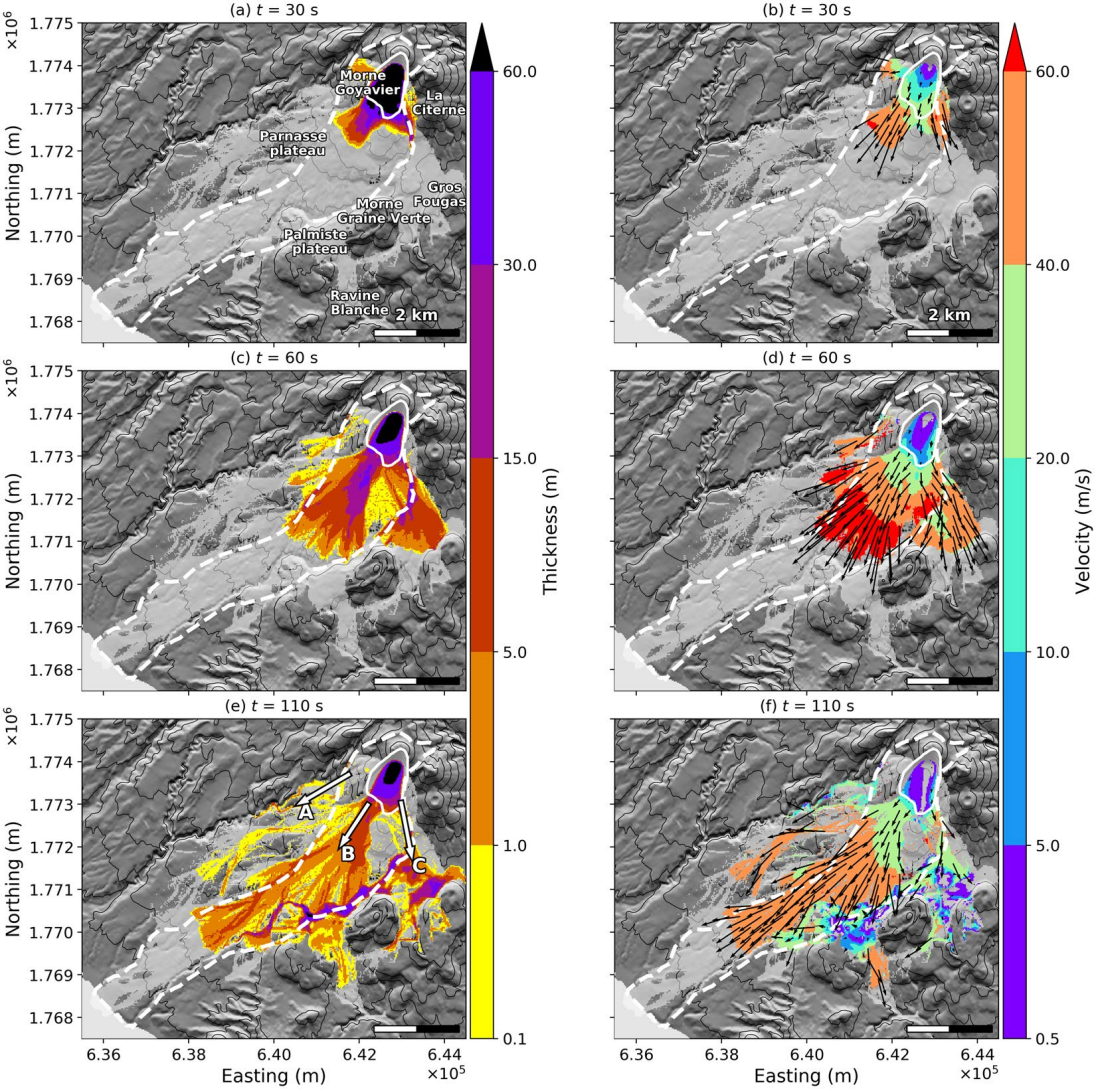
*topA2* simulation. Snapshots of flow thickness (**a**,**c**,**e**) and velocity (**b**,**d**,**f**) for the *topA2* scenario and *δ* = 7°, at *t* = 30 s (**a**,**b**), *t* = 60 s (**c**,**d**) and *t* = 110 s (**e**,**f**). Black arrows give the flow velocity direction. The light grey area features the flow path in the simulation, i.e. the total covered area. The white dashed line is the mapped extent of the 1530 CE collapse deposits^7–9^. The white plain line is the extent of the initial unstable volume in our simulation. Arrows A, B and C are the main flow directions, as in Fig. 1. The DEM is from IGN BDTopo, coordinates: WGS84, UTM20N. The contour interval is 100 m.

**Figure 6:**
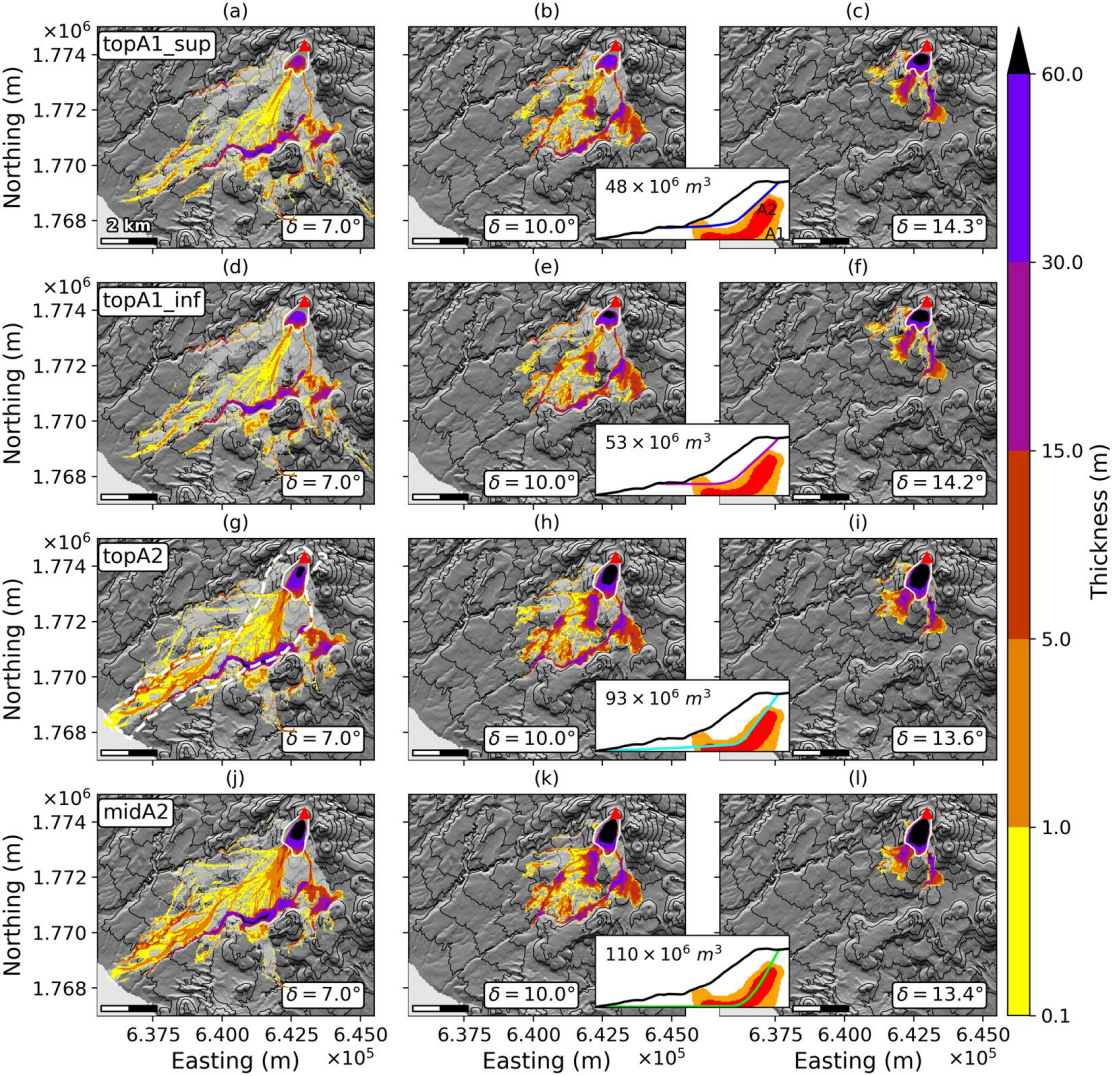
Final deposits for deep-rooted scenarios. *topA1_sup* (**a**–**c**), *topA1_inf* (**d**–**f**), *topA2* (**g**–**i**) and *midA2* (**j**–**l**) scenarios, with *δ* = 7° (**a**,**d**,**g**,**j**), *δ* = 10° (**b**,**e**,**h**,**k**) and the friction angle derived from Lucas’ law^52^ (**c**,**f**,**i**,**l**). Colorscale gives thicknesses in meters. The light grey area features the flow path in the simulation, i.e. the total covered area. The white plain line is the extent of the initial unstable volume. The red triangle marks the summit of La Soufrière volcano. The insert in each row displays the unstable volume and a profile of the scarp for each scenario: it is a copy of Fig. 3c where only the relevant scar has been kept. The DEM is from IGN BDTopo, coordinates: WGS84, UTM20N. The contour interval is 100 m.

The 1530 CE debris avalanche volume was estimated at 80 ± 40 × 10^6^ m^3^ ^7–9^. Its mapped extent, deduced from field observations^7–9^, is shown in Fig. 5 with the white dashed line. The estimated volume is consistent with our *topA2* scenario. In order for our modelled scenario to reach the sea like the 1530 CE collapse, we had to use a friction angle *δ* = 7°. In comparison, the friction angle derived from the empirical law of Lucas^52^ is *δ* = 13.6°. However, our best-fit friction angle is in good agreement with the value *δ* = 8° that was used to simulate with SHALTOP the debris flow part of the Mount Meager landslide in a previous study^55^. The dynamics from our simulation are given in Fig. 5 and the final deposits in Fig. 6(g). Three flow paths are clearly visible (Figs 1 and 5e, arrows A, B and C). Geographical references are shown in Fig. 1.

The first flow path (Figs 1 and 5e, arrow A) heads directly south-west between Morne Goyavier and the Rivière Noire ravine. It involves only small thicknesses (less than 1 meter 110 s after collapse, Fig. 5) but threatens the northern parts of Saint-Claude village. If the 1530 CE collapse had a similar behavior in this area, its thin deposits may have been easily eroded. Indeed, they have never been identified in the field.

The main feature, which is consistent with identified deposits of the 1530 CE event, is the material that spreads from Bain Jaunes (Fig. 1, ①) towards Basse-Terre (Figs 1 and 5e, arrow B). The flow is first partially channelled in the Ravine des Bains Jaunes and adjacent ravines (Fig. 1, ①). However, because it subsequently encounters no massive topographic barriers apart from the Parnasse lava flow to the north and the Galion ravine to the south, it produces deposits of limited thickness (up to 5 meters) with a large lateral extension. The flow is characterized by high velocities (70 m/s 60 seconds after collapse, 50 m/s 110 seconds after the collapse). The thickness of the distal deposits (up to 5 meters, Fig. 6(g)) is, furthermore, in agreement with the deposit thickness observed in the field (see supplementary information). The flow first spreads radially, but after 110 seconds the main flow front, about 500 meters wide, runs on the northern side of Galion River down to the sea which is reached after 200 seconds. This part of the flow is particularly fast and could generate jetted flow as described in the literature^61,62^ in historically observed rock avalanches (sturztroms). Such effects cannot, however, be modeled with SHALTOP, for which the flow is assumed to follow the topography closely.

The rest of the flow (Figs 1 and 5e, arrow C) is first channelized in the Galion river and in the Ravine de la Citerne (Fig. 1, ②), and bounces back and forth between the high walls of the ravines before filling and overspilling it (thickness up to 80 meters). It then spreads radially to the south of La Citerne scoria cone, into an area between the Morne Graine Verte and the Gros Fougas scoria cones further south. 110 seconds after collapse, the associated flow front has almost entirely stopped, except for a small patch that overpasses a notch in the Palmiste plateau, allowing it to flow to the south towards parts of Gourbeyre village. Although no desposits were found here for the 1530 CE event, older deposits have been identified in this area^7,8^.

### All scenarios

**Figure 7:**
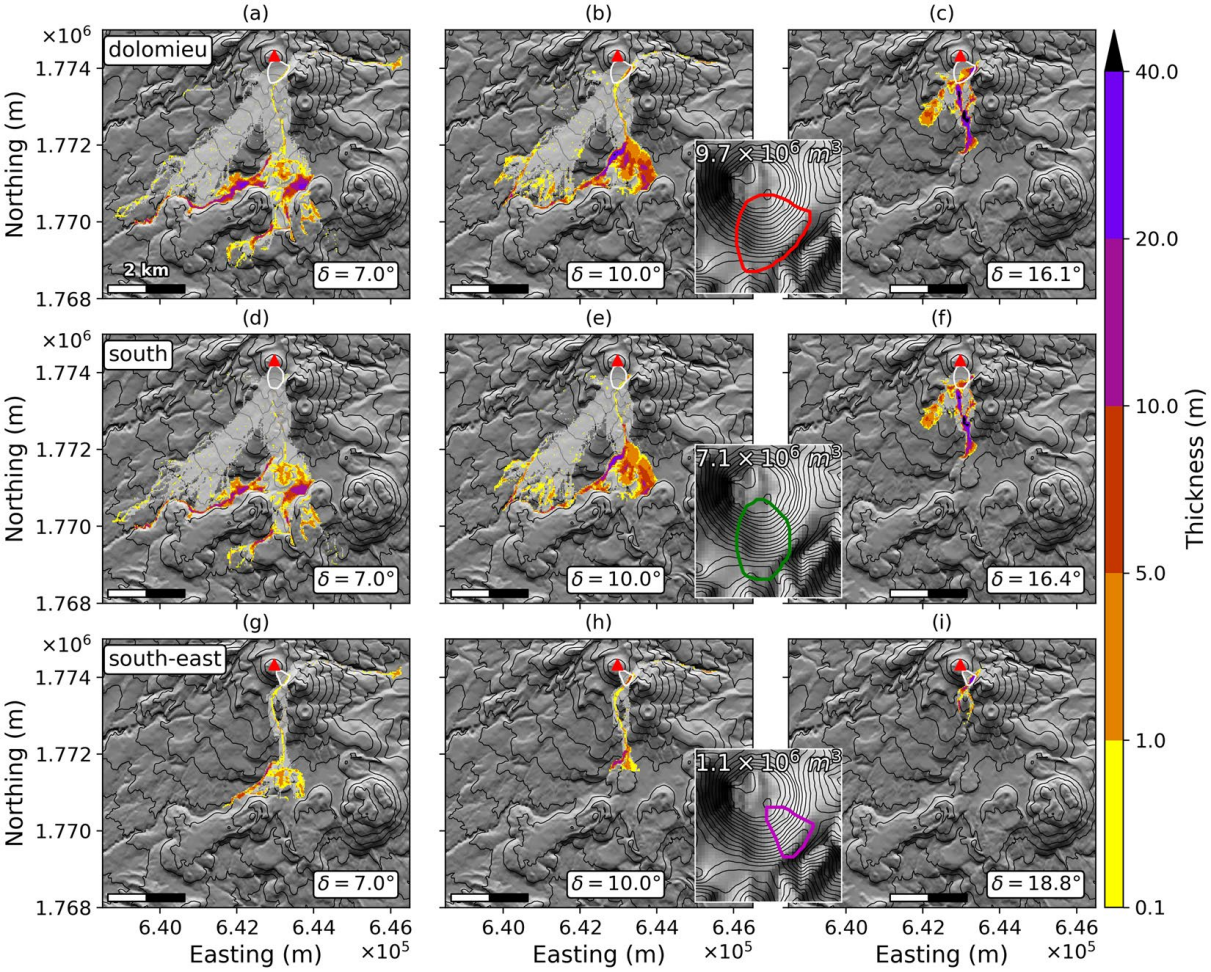
Final deposits for the superficial scenarios. *dolomieu* (**a**–**c**), *south* (**d**–**f**) and *south-east* (**g**–**i**) scenarii, with *δ* = 7° (**a**,**d**,**g**), *δ* = 10° (**b**,**e**,**h**) and the friction angle derived from Lucas’ law^52^ (**c**,**f**,**i**). The light grey area features the flow path in the simulation, i.e. the total covered area. The white plain line is the extent of the initial unstable volume. The red triangle marks the summit of La Soufrière volcano. The inserts in each row are close-ups on the dome showing the extent of the initial unstable volume and its volume for each scenario: they are copies of Fig. 4a,d and g. The DEM is from IGN BDTopo, coordinates: WGS84, UTM20N. The contour interval is 100 m for the main maps, and 20 m for the inserts.

Final deposits for all scenarios are displayed in Figs 6 and 7 for deep-rooted and superficial collapses respectively, with *δ* = 7°, *δ* = 10° and Lucas friction angles. Results are summarized in Table 1. The aforementioned pathways (Fig. 1, arrows A, B and C) can be identified in all scenarios.

The part of the modeled debris avalanches heading south-west towards Saint-Claude (Fig. 1, arrows A) does not cross the deep Rivière Noire canyon in any simulation. This natural barrier and the prominent Parnasse lava flow, that forms a massive topographic barrier on the eastern boundary of Saint-Claude thus channel the avalanche flow towards Saint-Claude and the northern quarters of Basse-Terre.

The second flow path (Fig. 1, arrow B) generates widespread deposits and is visible in all scenarios. For *δ* = 7°, it stops only a few hundreds meters away from the sea in the *topA1_sup* and *topA1_inf* scenarios, while the material enters the sea in *topA2* and *midA2* scenarios. In these four scenarios, still with *δ* = 7°, a small volume overtops the Palmiste plateau in its central part and enters Ravine Blanche.

The third flow path (Fig. 1, arrow C), generated by the material entering the Galion river, is present in all the scenarios. For friction angles above 10°, the debris avalanche overspills the Galion river but stops between Morne Graine vert and Gros Fougas in all scenarios. Only for *δ* = 7°, the flow comes to rest against the southern edge of the Palmiste lava plateau in the north-east periphery of Gourbeyre. In the three biggest scenarios, it enters Ravine Blanche.

The last flow path (Fig. 1, arrow D) is only seen in the *dolomieu* and *south-east* scenarios. This flow is generated by the material released in the active hydrothermal part of the dome between Fracture Lacroix and 8^*th*^ July 1976 fracture, as only the *dolomieu* and *south-east* scenarios include material in this area. The flow heads towards the east and is first mainly contained in the Rivière du Grand Carbet (Fig. 1). For *δ* = 7°, it then spreads in a flatter area about one kilometer after the second Chute du Carbet waterfall (Figs 1, ③) at the junction with the Grosse Corde River.

### Final deposits main characteristics

**Figure 8:**
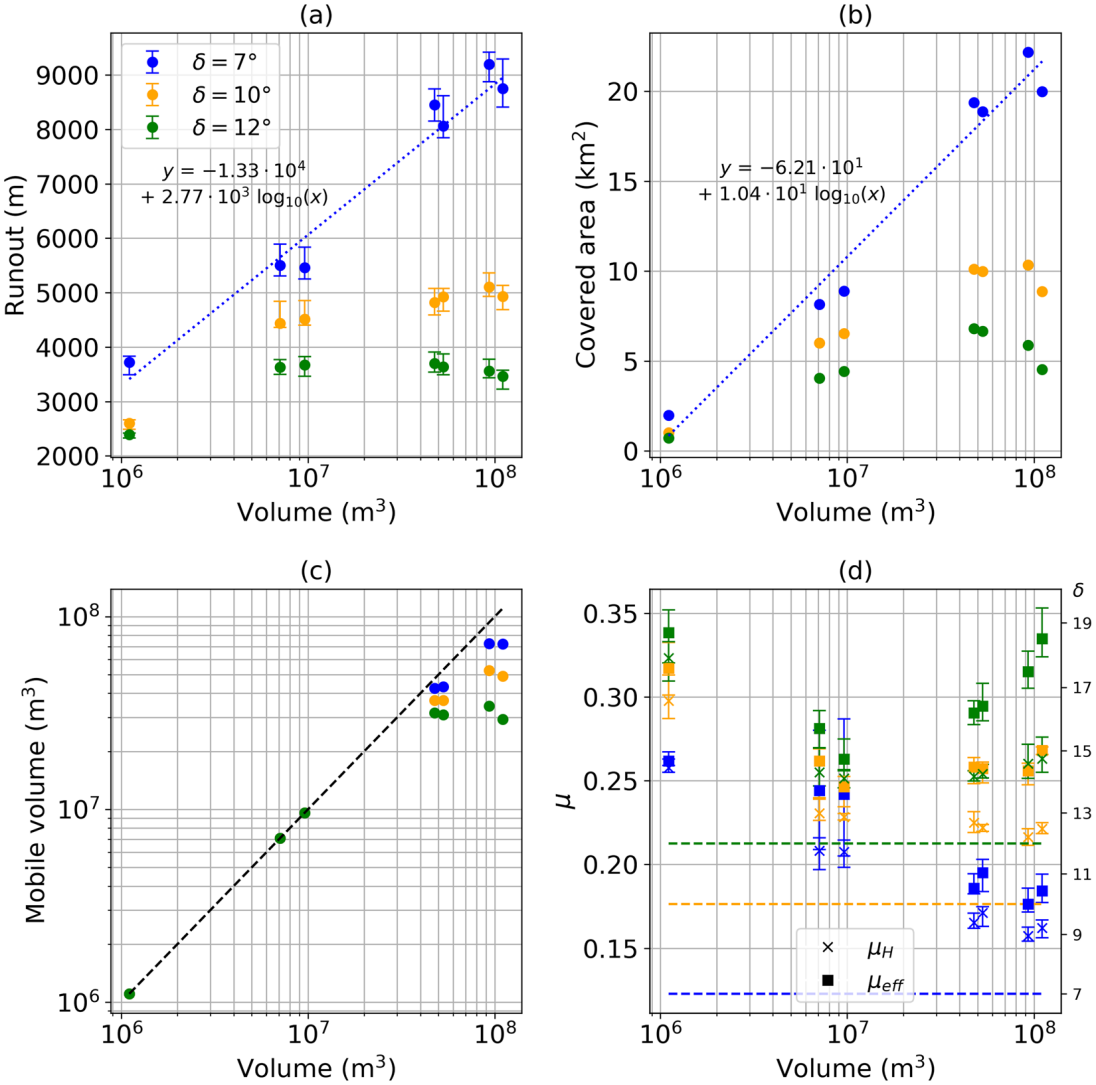
Main characteristics of debris avalanche deposits. Tested scenarios are categorized by unstable volume, with *δ* = 7° (blue), *δ* = 10° (orange) and *δ* = 12° (green). (**a**) Runout, i.e. maximum distance between the highest point in the scar prior to collapse and flow front. (**b**) Covered area. (**c**) Mobile volume, i.e. volume of the material leaving the scar (**d**) Heim’s ratio (crosses) and effective friction coefficient (squares). Values on the right y-axis are the angles *δ* in degrees, matching *μ* on the opposite axis, such that *μ* = *tan*(*δ*). Colored lines are the friction coefficients matching the tested friction angles. Error bars in (**a**) and (**d**) display the maximum, minimum and mean value derived following the methodology presented in the body of the text. The blue dashed lines in (**a**) and (**b**) are the best logarithmic fits derived for *δ* = 7°, with their matching equation indicated on the plots.

In Fig. 8 we summarize the main characteristics of the debris avalanche deposits resulting from the different modelled scenarios. For the seven collapse geometries, of varying volume, and for three friction angles (7°, 10°, 12°), we plot: runout (distance between scar highest point and deposit front), covered area, mobile volume (i.e. material leaving the scarp), Heim’s ratio (*μ*_*H*_) and effective friction coefficient (*μ*_*eff*_). The Heim’s ratio^61^ is defined as *μ*_*H*_ = *H*/Δ*L*′, where *H* and Δ*L*′ are respectively the difference in altitude and horizontal distance between the highest point of the original mass and the lowest point of the deposit. The effective friction coefficient was derived theoretically for a dam-break scenario^52^ and is defined by:

1 $${\mu }_{eff}=tan(\theta )+\frac{{H}_{0}}{\Delta L}$$

where *θ* is the mean slope angle along the flow course, *H*_0_ the maximum material thickness at the onset of the collapse and Δ*L* the length travelled by the flow front (see section Methods).

As expected, lower friction angles involve longer runouts (Fig. 8a) and greater deposit areas (Fig. 8b). For *δ* = 7° a consistent trend can be seen with increasing volume. However for *δ* = 12° and *δ* = 10°, the runout does not significantly vary between the *dolomieu*, *topA1_inf* and *topA1_sup* scenarios, while the deposit area is almost twice as large for *topA1_inf* and *topA1_sup* in comparison to *dolomieu*. This feature highlights the strong control of topography on the emplacement of the debris avalanche deposits. Topographic barriers slow down the flow front in the main flow direction and favor lateral spreading. For the four collapse geometries of largest volume, a significant part of the material remains blocked within the collapse structure due to its almost flat distal basal slope (Fig. 3b,c): for *midA2* and *δ* = 12°, only a volume of 30 × 10^6^ m^3^ leaves the structure while more than 100 × 10^6^ m^3^ is initially destabilized (Fig. 8c). The *topA2* geometry leads to a larger mobile volume even though it is more superficial. As a matter of fact, bigger collapses involve deeper scars, thus expanding the area of the collapse basal surface with a flat slope that cannot be overrun by a pure cohesionless collapse. This hence favors blockage of material within the structure and reduces the truly mobile portion of the collapse volume.

The Heim’s ratios of the modelled deposits are systematically lower than effective friction coefficients (Fig. 8d). Both overestimate the friction coefficient used in Shaltop. While Heim’s ratios seem to reach a constant value for unstable volume bigger than 10 × 10^6^ m^3^, effective friction coefficients show a sharp increase for *δ* = 10° and *δ* = 12°. It has been shown that *μ*_*eff*_ can be a better approximation of the real friction coefficient^52^ for simple coherent landslides but that does not seem to be the case here, for landslides made of multiple flows. These discrepancies illustrate the impossibility of using simple indicators whose theoretical validity stands only for simple coherent landslides, to describe more complex phenomena characterized by multiple channelizations and complex topographies.

## Discussion

In the case of the biggest collapse geometries, the strongly concave post-collapse topographies are associated to important deposit thicknesses. The shallow-water assumption is thus not valid as the thickness is not negligible in comparison to the initial flow extent, and we cannot expect our model to properly describe the initiation phase of the collapse. However, at least two reasons justify the use of shallow-water models. First, full 3D models demand significant computing resources and are time-consuming, while each of our simulations was run in less than 4 hours, which is a major advantage to carry out multiple simulations with various geometries and parameters for risk analysis. Secondly, previous studies have shown that shallow-water models can indeed reproduce real landslide deposits^52–54,63,64^.

Nevertheless complex topographies can favor threshold effects: for some paths to be taken by the debris avalanches, the scar geometry must have a minimum extent and/or define a minimum destabilized volume. For instance, including the material between the Lacroix and the 1956–8th July 1976 fractures in *dolomieu* and *south-east* scenarios enables some material to enter the Rivière du Grand Carbet, while no flow is modelled there in the *south* scenario whose eastern collapse boundary is only a few tens of meters west of Lacroix fracture. The overtopping of Palmiste lava Plateau, observed in the *topA2* scenario and not in the *midA2* scenario, is another example of such a threshold effect.

**Figure 9:**
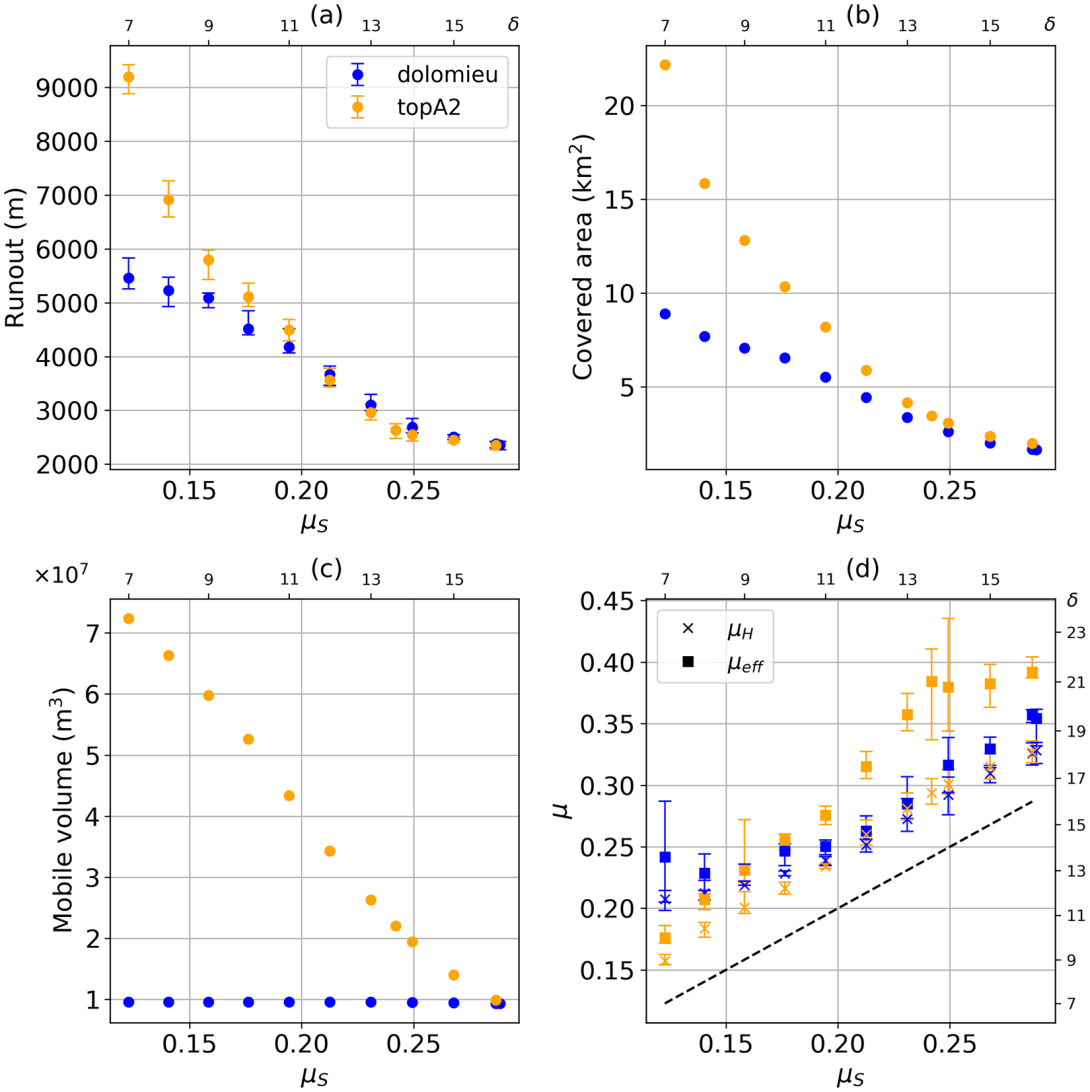
Main characteristics of debris avalanche deposits for the *dolomieu* (blue) and *topA2* (orange) scenarios. Varying friction coefficient are tested (from *δ* = 7° to *δ* = 16°). (**a**) Runout, i.e. maximum distance between the highest point in the collapse structure prior to collapse and flow front. (**b**) Covered area. (**c**) Mobile volume, i.e. volume of the material leaving the collapse structure (**d**) Heim’s ratio (crosses) and effective friction coefficient (squares). Values on the right y-axis in (**d**) and top x-axis are the angles *δ* in degrees, matching *μ* on the opposite axis, such that *μ* = *tan*(*δ*). Error bars in (**a**) and (**d**) display the maximum, minimum and mean value derived following the methodology presented in the body of the text.

Whether topographic barriers are crossed or not strongly depends on the volume of mobile material and on the friction coefficient. To illustrate this, simulations were run for the *dolomieu* and *topA2* scenarios with friction angles varying between 7° and 16° (i.e. friction coefficients between 0.12 and 0.29). Their characteristics are shown in Fig. 9. The *dolomieu* scenario’s scar is steep enough to enable all the material to flow (Fig. 9c). On the contrary, the truly mobile volume continually decreases in the *topA2* scenario as *μ*_*S*_ increases. For *μ*_*S*_ > 0.2 = *tan*(11.3°), the topography constrains the flow: although the mobile volume is bigger in *topA2* than in *dolomieu* scenario, runouts are similar (Fig. 9a) and spreading is more important in *topA2* (Fig. 9b). For *μ*_*S*_ < 0.2, both scenarios reach a smoother area west of the Palmiste Plateau. However in the *dolomieu* scenario, there is only little mobile material left, so that it becomes blocked in the Galion river and small valleys (Fig. 7). On the contrary, in the *topA2* scenario enough material is available to prevent confinement and the flow can propagate more easily (Fig. 6).

Finally, the difference between the friction coefficients *μ*_*S*_ used in the simulations, *μ*_*eff*_ and *μ*_*H*_ is clearly seen in Fig. 9d. In contrast to the case of landslides that dot not feature multiple channelizations^52^, *μ*_*H*_ over-estimates *μ*_*S*_ but for *μ*_*S*_ > 0.19 = *tan*(10.8°) the bias is almost constant and seems not to depend on the scenario (Fig. 9d).

The values of friction angles to be used in numerical simulation remain subject to debate. Lucas’ empirical law (that was derived for almost dry debris avalanches) yielded good fits in other volcanic contexts (Mount St. Helens and Soufrière Hills)^64^ where seismic data was used to constrain the simulation parameters. However in our case the value *μ*_*S*_ = *tan*(7°) used to reproduce an analogue of the 1530 CE event is much lower than the Lucas empirical value of *μ*_*eff*_ = *tan*(13.6°). This suggests a strong mobility of the debris avalanche that could be explained by the presence of water. Indeed, such a low friction angle (*δ* = 8°) was needed with the same SHALTOP model to reproduce debris flows^55^. This is consistent with the 1530 CE event that is characterized in the field by deposits^7,8^ with a well-developed muddy textural facies (see supplementary information). The transition between a debris avalanche and debris flow emplacement mechanism occurs about 5 km from the source and about half-way along the total runout distance. Water could thus have a prominent role in controlling the dynamics of future partial flank collapse and debris avalanche mobility, particularly for the deep rooted landslides cutting through the highly conductive fluid-saturated bodies A1 and A2. For instance, more than 13% of the collapsing volume of the *midA2* scenario lies within these bodies (Table 1). But there are numerous other sources of water in the La Soufrière of Guadeloupe volcano, such as perched aquifers inside the lava dome^32^, shallow depth reservoirs of hydrothermal fluids^27,32,33^, depressurized deep rising hydrothermal fluids^12,30^, rivers and extreme rainfall. This is typical of the volcanic context. For instance, the debris avalanche of August 2012 on Tongariro^65,66^ and the 1998 debris avalanche on Casita volcano^24,67,68^ both initiated as debris avalanches but later transformed into more mobile and devastating debris flows, mostly in their distal part.

## Conclusion

Our modelling of geologically and geophysically constrained partial collapse scenarios at La Soufrière of Guadeloupe provides key insights on the propagation dynamics and controlling factors of the resulting debris avalanches. Back calibration of the last flank collapse in 1530 CE and field evidence from deposit textures suggest such an event could be highly mobile due to the presence of water in the flow. Multiple simulations of debris avalanches were carried out with various initial geometries constrained by morphological and geophysical data, as well as different friction angles. Four main trends were identified for the flows as they are channelled by the topography, with two different dynamics. Part of the flow fills the ravines and stops quickly while another part reaches open areas where it spreads with high velocities and limited thicknesses. We show that the initial collapse geometry plays a major role in our model in retaining material upslope and thus controlling the volume effectively leaving the collapse structure. For the biggest collapse geometries, friction coefficients below 0.2 (i.e. friction angles below 11°) increase the mobile volume and favor overtopping of topographic barriers by the flow.

In these simulations, the northern and eastern parts of Saint-Claude are the most exposed inhabited areas. They could be impacted even by shallow small-volume partial dome collapse (our dolomieu and south scenarios) if water is incorporated in the debris avalanche, increasing its mobility. In the case of a major dome collapse, the mixing of the altered material with perched ground water and hydrothermal fluids could threaten Basse-Terre, with the propagation of a relatively thin but rapid flow. Gourbeyre is at first sight well protected by the Palmiste plateau but massive collapse and/or mobile flows could threaten its eastern periphery. However, in the most probable event of a small collapse from the most active part of the dome (our *south-east* scenario), the material should be mainly confined in the ravines and impact only remote areas. Nevertheless, all the material accumulated in the ravines could form temporary dams and be easily remobilized as debris flows or mud flows long after the initial landslide, thus endangering urban areas all along the rivers. The Rivière des Pères, the Galion river, the Rivière du Grand Carbet and the Rivière Grand Anse (heading to the south towards Trois Rivières) would be particularly exposed.

Given the current unrest of La Soufrière of Guadeloupe volcano, the work initiated in this study must be continued in order to improve risk assessment associated with a partial dome collapse. In particular, a limit stability analysis would help constrain the unstable volumes in the dome. Numerous simulations randomly sampling a range of model parameters could also be developed to produce probabilistic debris avalanche innundation maps.

## Methods

### Scar geometries

The three superficial collapse structures were constructed using an interface specifically developed for that purpose. Starting from a 25-meter DEM of the intact lava dome, the surface to be modified was defined from geological constraints. The *z*-value of a series of control points within this surface was then manually modified, the rest being interpolated with the MATLAB TriScatteredInterp function and the natural interpolation method. The collapse structure was then smoothed with a moving average.

The four deep rooted collapse structures were constructed with a similar approach. Control points were given along 5 longitudinal profiles, one being the profile displayed in Fig. 3c (used as reference). 30 points were then generated for each profile along a Beziers curve passing through all control points. Finally, the 150 resulting points (plus some manually added points to refine the interpolation) were used to interpolate the scar with a multiquadric radial basis function.

In both cases, the thickness of the initial unstable volume was given in each point of coordinates (*x*, *y*) by:

2 $$h=\,\cos (\theta )({z}_{init}-{z}_{scar}),$$

where *z*_*init*_ is the altitude of the initial DEM, *z*_*scar*_ the altitude of the DEM including the collapse structure, and *θ* the local slope angle. The volume of destabilized material is computed with:

3 $$V=\sum _{i,j}({z}_{init}(i,j)-{z}_{scar}(i,j))dx\,dy,$$

where *dx* = *dy* = 25*m* and the indexes (*i*, *j*) indicate the position on the 25-meter DEM.

### Computation of runout, Heim’s ratio and effective friction coefficient

**Figure 10:**
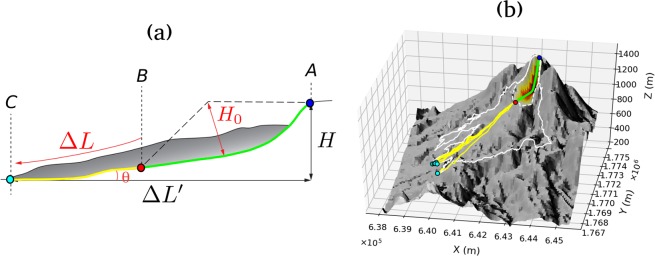
Computation of *μ*_*H*_ and *μ*_*eff*_. (**a**) Landslides metrics in 2D (after^52^). *A* (blue point) and *B* (red point) are respectively the highest and lowest points of the initial mass. *C* (cyan point) is the flow front position. *H*_0_ is the initial mass maximum thickness. *H* and Δ*L*′ are respectively the difference in altitude and horizontal distance between *A* and *C*. Δ*L* is the length traveled by the front flow, i.e. the length of the yellow curve. *θ* is the mean local slope between *A* and *C*, i.e. the mean local slope of the joint yellow and green curves. The Heim’s ratio is *μ*_*H*_ = *H*/Δ*L*′, and the effective friction coefficient is *μ*_*eff*_ = *tan*(*θ*) + *H*_0_/Δ*L*. (**b**) Landslides metrics in 3D, for the *topA2* scenario with *δ* = 10. Color code of lines and points matches (**a**). The white line is the deposit extent, the dashed white line is the collapse scar extent. Colorscale in the collapse geometry matches the initial mass thickness (from yellow to red). Points *A* and *B* are uniquely defined. Points C are chosen as explained in the main body of the text. The DEM is from IGN BDTopo, coordinates: WGS84, UTM20N.

The metrics we use are shown in Fig. 10. For a given simulation, we first derive the map of the maximum flow thickness for the entire simulation and draw the contour line corresponding to a 10 cm thickness. We then compute the geographical distance Δ*L*′ to the upper point of the collapse structure (Fig. 10, point *A*) along this line and identify all local maximums. We keep only the point with the global maximum distance *d*_*max*_, and all points further than 0.9*d*_*max*_ (Fig. 10, points *C*). We then derive straight profiles along topography from *A* to *C*, passing through the lowest point of the collapse geometry (Fig. 10, point *B*). Runout (i.e. Δ*L*′), *μ*_*H*_ and *μ*_*eff*_ are computed for all these profiles, yielding a variability estimation that is displayed with error bars in Figs 8 and 9.

## Supplementary information

Supplementary Information
