# Supplementary material for: Modeling of partial dome collapse of La Soufrière of Guadeloupe volcano: implications for hazard assessment and monitoring
Source: Sci Rep. 2019 Sep 11;9:13105. doi: 10.1038/s41598-019-49507-0 (PMC6739312; doi:10.1038/s41598-019-49507-0)
Supplement: Supplementary file 1 — Supplementary Information [file 41598_2019_49507_MOESM1_ESM.pdf]

# Supplementary information
## Modeling of partial dome collapse of La Soufrière of Guadeloupe volcano: implications for hazard assessment and monitoring

Marc Peruzzetto[1,2*], Jean-Christophe Komorowski[1], Anne Le Friant[1], Marina Rosas-Carbajal[1], Anne Mangeney[1], and Yoann Legendre[3]

[1] Université de Paris, Institut de physique du globe de Paris, CNRS, F-75005 Paris, France
[2]BRGM, Orléans, France
[3]BRGM, Guadeloupe, France
[*]peruzzetto@ipgp.fr

## A Field evidence of the 1530 CE debris avalanche

The field evidence for the 1530 CE debris avalanche of La Soufrière, collected between 1994 and 2008, has been partially discussed in previous works, such as [Komorowski, 2008], [Boudon et al., 2008] and [Legendre, 2012]. Figure A.1 was produced from a compilation and new interpretation of these data in terms of location, thickness and textural facies of the identified deposits. The co-existence of deposits showing a typical debris avalanche facies with those showing a muddy debris-flow facies is striking and highlights both the complexity and mobility of the overall flow. Although localized deposits with a debris flow facies were found only 850 meters away from the summit in Ravine Tarade, all other occurrences of debris flow facies are located between a distance of 5.6 km from source in the north-eastern periphery of Basse-Terre and the Caribbean sea. The occurrences of deposits with debris avalanche facies furthest from source are located north of the Palmiste Plateau about 5.6 km from source. The 1530 CE partial edifice collapse may thus initially emplaced a debris avalanche with only localized debris flows in the vicinity of the volcano. After travelling between 3.6 and 5.6 kilometers south-west, it transformed into a massive thick debris flow given its initial elevated water content and reached the sea. A precise comparison with our simulation is complex due to the uncertainty on the initial conditions of the 1530 CE debris avalanche, and on the topography that may have changed since the event. In particular, the 1530 CE debris avalanche deposits seem to be thicker near the mouth of The Galion river than in our simulation. However in the area of Basse-Terre, the orders magnitude of mapped and simulated thicknesses are similar.

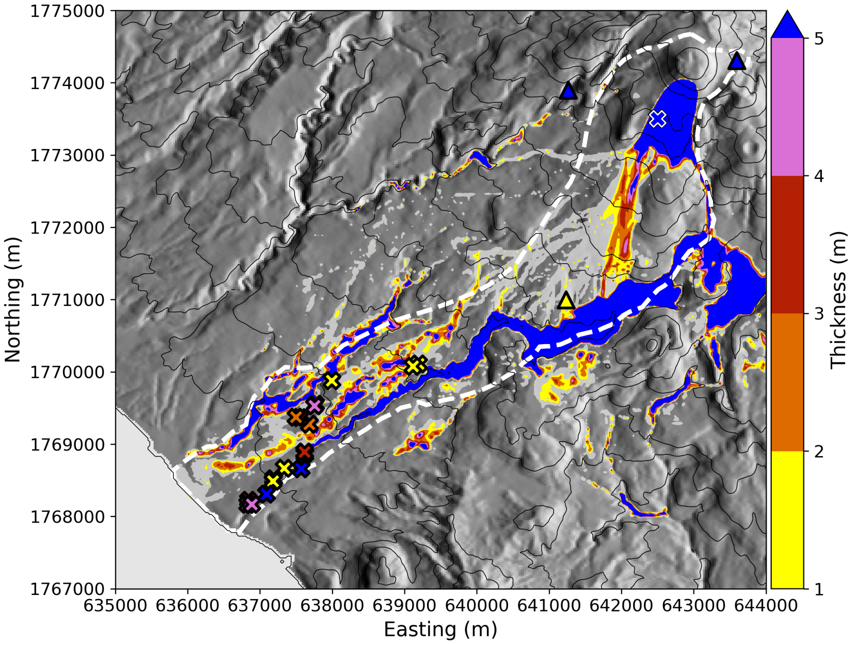


Figure A.1: **Comparison between observed and simulated deposits for the 1530 CE debris avalanche**. Deposits identified on the field with debris avalanche facies are marked with triangles, and deposits with debris flow facies are marked with crosses. The color of the symbols indicates the thickness of the deposits. The white dashed line is the estimated extent of the deposits. The simulated deposits of the *topA2* scenario with $\delta = 7°$ are displayed with the same colorscale. The light grey area matches the flow path in the simulation. The DEM is from IGN BDTopo, coordinates: WGS84, UTM20N. The contour interval is 100 m.

# References

[Boudon et al., 2008] Boudon, G., Komorowski, J.-C., Villemant, B., and Semet, M. P. (2008). A new scenario for the last magmatic eruption of La Soufrière of Guadeloupe (Lesser Antilles) in 1530 A.D. Evidence from stratigraphy radiocarbon dating and magmatic evolution of erupted products. *Journal of Volcanology and Geothermal Research*, 178(3):474–490.

[Komorowski, 2008] Komorowski, J.-C. (2008). Du volcan au pyroclaste: une approche pluridisciplinaire de la compréhension des processus éruptifs et de l'évaluation des aléas. Mémoire d'Habilitation à Diriger la Recherche, Université René Diderot Paris 7.

[Legendre, 2012] Legendre, Y. (2012). *Reconstruction fine de l'histoire éruptive et scenarii éruptifs à la soufrière de Guadeloupe : vers un modèle intégré de fonctionnement du volcan.* thesis, Paris 7.
